# Measuring repeatability of dynamic contrast-enhanced MRI biomarkers improves evaluation of biological response to radiotherapy in lung cancer

**DOI:** 10.1007/s00330-024-10970-7

**Published:** 2024-08-09

**Authors:** Nivetha Sridharan, Ahmed Salem, Ross A. Little, Maira Tariq, Susan Cheung, Michael J. Dubec, Corinne Faivre-Finn, Geoffrey J. M. Parker, Nuria Porta, James P. B. O’Connor

**Affiliations:** 1 Clinical Trials and Statistics Unit, The Institute of Cancer Research, London, UK; 2 Division of Radiotherapy and Imaging, The Institute of Cancer Research, London, UK; 3 Division of Cancer Sciences, University of Manchester, Manchester, UK; 4 Department of Anatomy, Physiology and Biochemistry, Faculty of Medicine, The Hashemite University, Zarqa, Jordan; 5 Christie Medical Physics and Engineering, The Christie NHS Foundation Trust, Manchester, UK; 6 Clinical Oncology, The Christie NHS Foundation Trust, Manchester, UK; 7 Bioxydyn Ltd, Manchester, UK; 8 Centre for Medical Image Computing, Department of Medical Physics and Biomedical Engineering, University College London, London, UK; 9 Radiology Department, The Christie NHS Foundation Trust, Manchester, UK

**Keywords:** Biomarkers, Magnetic resonance imaging, Lung neoplasms, Statistics

## Abstract

**Objectives:**

To measure dynamic contrast-enhanced magnetic resonance imaging (DCE-MRI) biomarker repeatability in patients with non-small cell lung cancer (NSCLC). To use these statistics to identify which individual target lesions show early biological response.

**Materials and methods:**

A single-centre, prospective DCE-MRI study was performed between September 2015 and April 2017. Patients with NSCLC were scanned before standard-of-care radiotherapy to evaluate biomarker repeatability and two weeks into therapy to evaluate biological response. Volume transfer constant (*K*^trans^), extravascular extracellular space volume fraction (*v*_e_) and plasma volume fraction (*v*_p_) were measured at each timepoint along with tumour volume. Repeatability was assessed using a within-subject coefficient of variation (wCV) and repeatability coefficient (RC). Cohort treatment effects on biomarkers were estimated using mixed-effects models. RC limits of agreement revealed which individual target lesions changed beyond that expected with biomarker daily variation.

**Results:**

Fourteen patients (mean age, 67 years +/− 12, 8 men) had 22 evaluable lesions (12 primary tumours, 8 nodal metastases, 2 distant metastases). The wCV (in 8/14 patients) was between 9.16% to 17.02% for all biomarkers except for *v*_p_, which was 42.44%. Cohort-level changes were significant for *K*^trans^ and *v*_e_ (*p* < 0.001) and tumour volume (*p* = 0.002). *K*^trans^ and tumour volume consistently showed the greatest number of individual lesions showing biological response. In distinction, no individual lesions had a real change in *v*_e_ despite the cohort-level change.

**Conclusion:**

Identifying individual early biological responders provided additional information to that derived from conventional cohort cohort-level statistics, helping to prioritise which parameters would be best taken forward into future studies.

**Clinical relevance statement:**

Dynamic contrast-enhanced magnetic resonance imaging biomarkers *K*^trans^ and tumour volume are repeatable and detect early treatment-induced changes at both cohort and individual lesion levels, supporting their use in further evaluation of radiotherapy and targeted therapeutics.

**Key Points:**

- *Few literature studies report quantitative imaging biomarker precision, by measuring repeatability or reproducibility*.
- *Several DCE-MRI biomarkers of lung cancer tumour microenvironment were highly repeatable*.
- *Repeatability coefficient measurements enabled lesion-specific evaluation of early biological response to therapy, improving conventional assessment*.

## Introduction

Functional imaging enables the detection and monitoring of early changes in the tumour microenvironment induced by targeted therapies [1] and conventional chemoradiotherapy [2]. Examples include measurements of elevated glucose metabolism with ^18^F-fluorodeoxyglucose (FDG) positron emission tomography-computed tomography (PET-CT) [3], altered cellular structure and density with diffusion-weighted imaging [4], presence of hypoxia with oxygen-enhanced magnetic resonance imaging (MRI) or hypoxia PET methods [5, 6], and altered perfusion with dynamic contrast-enhanced MRI (DCE-MRI) [7]. In these techniques, early change in measurements following therapy can be used to identify new drug targets (such as metabolism or angiogenesis) and to quantify the ‘biological response’ to these therapies. Furthermore, these biological responses may predict subsequent ‘clinical response’ as defined by RECIST and other evaluation systems [8].

Measurements derived from MRI and other modalities are termed ‘imaging biomarkers’. In oncology, these data provide non-invasive, serial, three-dimensional, whole tumour assessment of the microenvironment and can demonstrate differential biological responses in multiple lesions within the same patient [9, 10]. When used to full potential, such data can provide substantial insight into tumour biology and therapy mechanism of action [1, 2]. However, few imaging biomarkers are translated into tools used for decision-making in the clinic [9].

Measurement precision is an under-reported but important step in biomarker translation [11]. It can be quantified by single-centre repeatability of a biomarker value [9] and these data can be used for two purposes. Firstly, they provide a literature benchmark to assess the technical performance of the biomarker against other studies. Secondly, the size of the treatment effect can be compared with measures of repeatability to determine if therapy-induced change in a biomarker exceeds the amount expected through daily variation [12, 13].

In this study, we evaluated measures of DCE-MRI biomarker repeatability in patients with non-small cell lung cancer (NSCLC) treated with radiotherapy-based regimens. DCE-MRI is a technique that has been widely applied in the clinical trial setting to evaluate changes in blood flow and permeability, induced by anti-vascular drugs [7] and has also been used to assess similar changes in the tumour microvasculature following standard-of-care treatment. Radiotherapy plays an important role in the management of nearly all patients with lung cancer [14]. Although DCE-MRI is known to be feasible in NSCLC patients [15], have acceptable inter and intra-rater reliability [16] and be capable of detecting microenvironmental change following (chemo)-radiotherapy [17], the repeatability of these biomarkers is unknown in this setting.

The primary aim was to estimate DCE-MRI biomarker repeatability in NSCLC. The secondary aims were to demonstrate how repeatability estimates can identify which target lesions exhibit early biological response and to compare this assessment of therapeutic response with those obtained from conventional cohort statistics.

## Materials and methods

Institutional review board approval was obtained at The Christie NHS Foundation Trust (reference: 15/NW/0264). All patients provided written informed consent.

### Study design

Patients with NSCLC eligible for curative-intent radiotherapy either alone or with induction or concurrent chemotherapy were recruited prospectively from a single-centre between September 2015 and April 2017. Inclusion criteria were age ≥ 18 years old, histology or cytology confirmed NSCLC, Eastern Cooperative Oncology Group performance score of ≤ 2 and serum creatinine < 120 μmol/L. Coexistent chronic obstructive pulmonary disease was allowed only where patients had a forced expiratory volume in 1 s greater than 1 litre, or > 40% predicted value on lung function testing. Patients with distant metastasis were included only if eligible for curative-intent therapy. Exclusion criteria were standard contraindications to MRI.

Patients underwent DCE-MRI scanning at baseline prior to treatment. A retest, and repeat baseline were performed when logistically possible. Radiation treatment was delivered with intensity-modulated radiotherapy on a linear accelerator (55 Gy in 20 daily fractions or 60 Gy in 30 daily fractions). Patients underwent a final DCE-MRI scan at 2 weeks into therapy [18].

### MRI data acquisition

MRI data were acquired on a 1.5-T whole body scanner (Philips Achieva, Philips Medical Systems) using Sense XL Torso (DCE-MRI) coils. Initial localiser and anatomic scans were performed. Then DCE-MRI data were acquired in the coronal plane.

Pre-contrast *T*_1_ was estimated using a coronal 3D *T*_1_ Fast Field Echo variable flip angle protocol (repetition time 3.3 ms; echo time 1.43 ms; *α* = 2°/4°/7°/10°). The field of view was 450 × 450 × 205 mm. 41 slices were acquired (5 mm thickness). The in-plane resolution was 4.69 × 4.69 mm (from a 96 × 96 acquisition matrix). The dynamic series had the same *T*_1_-weighted acquisition, matched for the field of view with a 10° dynamic acquisition over 75 timepoints (temporal resolution: 4.9 s). After 6 measurements, a bolus of 0.05 mmol/kg gadoterate meglumine (Dotarem®, Guerbet) was injected at 1.5 mL/s followed by saline into a forearm vein using a Spectris MR (Medrad Inc) power injector.

### MRI biomarker derivation

The tumour region of interest (ROI) was defined by a board-certified radiologist (J.P.B.O’C., 18 years’ experience), using Jim 6 software (Xinapse systems) on coronal postcontrast gadolinium *T*_1_-weighted images. Lesions measuring 2.5 cm or greater in the largest axial dimension were included. Non-linear diffeomorphic image registration (stnava.github.io/ANTs) corrected for breathing and patient motion during the dynamic series. Tumour ROIs were then transferred to dynamic series and checked for spatial accuracy. Lesion whole tumour volume (WTV) was calculated in cm^3^. Quality control steps were performed by an independent observer (R.A.L.), including checking for protocol adherence, a positive control assessment of *T*_1_, the presence of a valid uptake curve post-gadolinium in the tumour and the presence of motion following motion correction.

An input function was measured for each patient from the thoracic aorta. The volume transfer constant (*K*^trans^), fractional volume of the extravascular extracellular space (*v*_*e*_), and fractional blood plasma volume (*v*_*p*_) were calculated from the extended Tofts version of the Kety tracer kinetic model [19]. The model-free initial area under the gadolinium contrast agent concentration-time curve up to 60 s post-injection (*IAUC*_60_) was also calculated [20]. All parameters were calculated voxel-wise for each individual tumour. Median values of *IAUC*_60_, *K*^trans^, *v*_*e*_ and *T*_1_, and the mean *v*_*p*_ across voxels were determined from the enhancing portion of each tumour ROI [21], defined by *IAUC*_60_ > 0 (one-sided paired sample *t*-test, *p* < 0.05) [22]. Analysis was performed using in-house software [23].

### Evaluation of biomarker distributions

Data distributions of each MRI biomarker (WTV, *T*_1_ and DCE-MRI metrics) both at pre-treatment (hereafter termed ‘baseline’) and at week 2 were evaluated with histograms. Normality was determined by examining the cohort distribution of average values at baseline (mean or median). The Shapiro-Wilks test was used to identify departures from the normal distribution. Parameters exhibiting non-normal distribution and/or evident variability in measurement error (see below) underwent a logarithmic transformation.

### Estimates of biomarker repeatability

Biomarker repeatability refers to the magnitude of its measurement error under a set of repeatable conditions and was estimated following recommendations by the Quantitative Imaging Biomarker Alliance [11]. Details of the statistical model specification can be found in the Supplementary materials and methods.

In brief, Bland-Altman plots [24] were used to represent the mean of the replicates for each lesion and compare their difference, thus illustrating trends in the variability of the biomarker over the range of measurement values. When the variability was not constant, a logarithmic transformation of the data was performed. This variability is designated as within-lesion variability (noted below by ${\sigma }_{w}^{2}$), and repeatability metrics are derived from it by specifying and fitting a random effects model [25].

The repeatability coefficient (RC) is defined as the least significant difference between any two repeated measurements taken under identical conditions at a two-sided significance of $\alpha =$ 0.05 [11]. Parameter changes exceeding the +RC threshold or falling below the −RC threshold (limits of agreement, LOA) can be regarded as real changes, not attributed to measurement error, with a 95% confidence level (hereafter the term ‘real change’ is used for this specific interpretation).

When normal distributions cannot be assumed or the within-lesion variance varies with the magnitude of the biomarker, the within-lesion coefficient of variation (wCV) is usually reported, defined as the ratio of the within-lesion standard deviation by the mean of the replicates and expressed in %.

We calculated the asymmetric LOA: where MRI biomarkers are lognormally distributed, the standard deviation of the parameter varies proportionally with the mean, and the ${wCV}$ can be also expressed as

$${wCV}=\sqrt{{e}^{{\sigma }_{{{{\mathrm{ln}}}}}^{2}}-1}$$

where ${\sigma }_{{{{\mathrm{ln}}}}\,}^{2}$ is the within-lesion variance of the log-transformed data [13]. In the logarithm scale, ${RC}$ can be calculated as ${\widehat{{RC}}}_{{{{\mathrm{ln}}}}}=1.96\sqrt{2{\hat{\sigma }}_{{{{\mathrm{ln}}}}}^{2}}$ [13]. This provides asymmetric LOA when back-transformed to the original scale:

$${{RC}}_{L}=(\exp \left(-{\widehat{{RC}}}_{{{{\mathrm{ln}}}}}\right)-1)\,{{{{\rm{and}}}}}\,{{RC}}_{U}=(\exp \left(+{\widehat{{RC}}}_{{{{\mathrm{ln}}}}}\right)-1)$$

### Biomarker estimates of cohort mean treatment effect with mixed-effects modelling

For each biomarker, response to therapy was examined in the cohort by calculating percentage change from baseline to week 2 (on the log-transformed data), by fitting a mixed-effects model where random effects account for clustering of lesions within the patients, and scan timepoints (pre-treatment, week 2) incorporated as fixed effects (see Supplementary materials and methods). A significant treatment effect over time is determined when the parameter for the fixed effect is estimated to be significantly different from zero. For patients with two baseline scans, the average value of the two scans determined the baseline biomarker value.

### Identifying individual lesions exhibiting significant treatment effect

For each MRI biomarker, and each lesion, waterfall plots were used to represent the percentage change from the pre-treatment values to week 2 [7]. We determined the frequency of individual lesion changes exceeding the asymmetric RC LOA obtained from the repeatability analyses. In addition, where available, literature-based thresholds were applied. We used published thresholds of 40% change in either direction for median *K*^trans^ [26]. For WTV we derived thresholds based on 1D RECIST [8] and 2D World Health Organisation (WHO) size-based changes [27], with an increase of 72.8% and a decrease of 65.7% indicating the change (details in Supplementary materials and methods).

### Statistical analyses

All statistical tests were performed using Stata/BE 17.0 (StataCorp LLC). A significance level of 0.05 was used for all tests. A career statistician (N.P.) was the statistics guarantor.

## Results

**Fig. 1:**
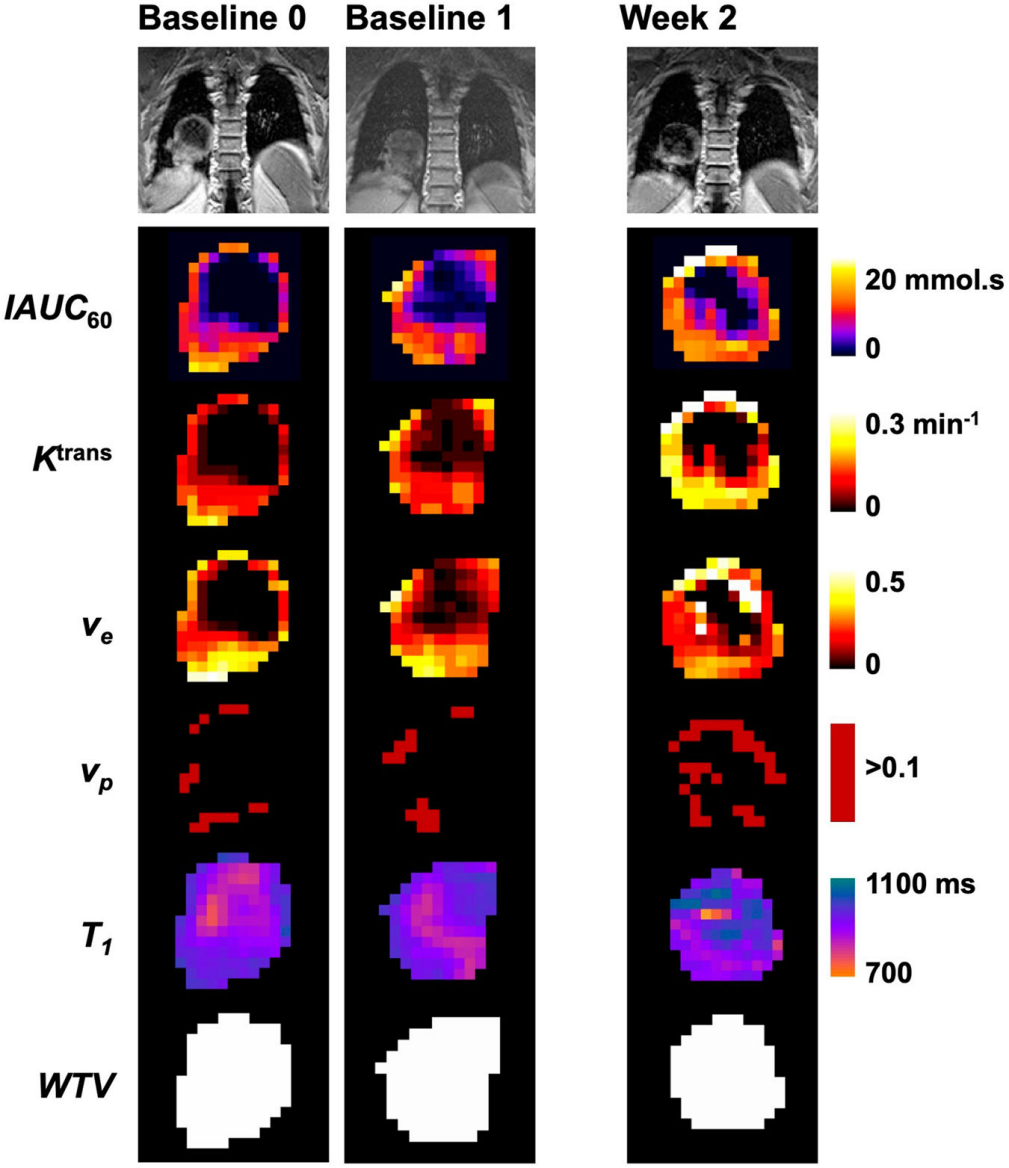
Example images of primary lung cancer in a patient who underwent MRI on all three occasions

Overall, 14 patients were recruited (mean age, 67 years +/− 12, 8 men). They had 22 tumours between them, comprising 12 primary lung cancers, 8 metastatic lymph nodes in the thorax and 2 distant metastases within the field of view. Details of the patients, their demographics and their lesions are presented in Table 1. All patients had either completed induction chemotherapy or were not eligible for chemotherapy, except for patient 4 who underwent concurrent chemoradiotherapy. Therefore, changes in MRI biomarkers were considered largely due to radiation. Sample images are shown in Fig. 1.

**Table 1:** Patient demographics, disease stage, treatment, and collection of MRI data

ID	Index lesion number	Sex	Age	Histology	TNM stage	RT dose (Gy)	Chemo-therapy	Target lesions	Extra baseline	Pre-treatment baseline	Week 2
1	1	M	46	Squamous cell	T3N2M1b	55	Induction	T	✓	✓	✓
2	2, 3, 4	F	78	Adenocarcinoma	T3N2M1b	55	Induction	T, N, M	✓	✓	✓
3	5, 6, 7	F	70	Squamous cell	T3N2M0	55	Induction	T, N, N	✓	✓	✓
4	8, 9	F	71	Adenocarcinoma	T3N2M0	66	Concurrent	T, N	✓	✓	✓
5	10	M	80	Squamous cell	T3N2M0	55	None	T		✓	✓
6	11	F	68	Adenocarcinoma	TxN2M0	55	None	N	✓	✓	✓
7	12	F	78	Adenocarcinoma	T4N3M0	60	Induction	T		✓	✓
8	13	M	62	Adenocarcinoma	T3N2M0	55	Induction	T		✓	✓
9	14	F	47	Squamous cell	TxN2M0	55	Induction	N		✓	✓
10	15	M	55	Squamous cell	T4N3M0	55	Induction	T		✓	✓
11	16	M	78	Adenocarcinoma	T1N0M0	55	None	T		✓	✓
12	17, 18	M	57	Squamous cell	T3N0M1b	55	Induction	T, M	✓	✓	✓
13	19, 20, 21	M	82	Adenocarcinoma	T2N2M0	55	None	T, N, N	✓	✓	✓
14	22	M	76	Adenocarcinoma	T4N2M0	none	Induction	T	✓	✓	

Stage was based on *T* primary tumour, *N* nodal tumour, *M* distant organ metastasis

### Evaluation of biomarker distributions

**Fig. 2:**
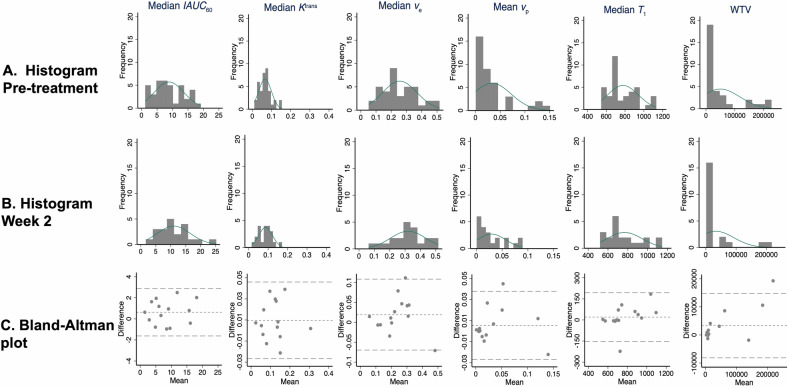
Histogram distributions of the cohort parameter values for each of the six MRI biomarkers at (**A**) pre-treatment baseline and (**B**) week 2 of treatment. Measurement error of the test-retest replicates pre-treatment is shown in **C** with Bland-Altman plots, where the top and bottom dotted lines indicate LOA

Histograms revealed skewed, distributions of all MRI biomarker parameters that deviated from normality except for pre-treatment median *IAUC*_60_ and pre-treatment median *v*_*e*_ (Fig. 2A, B and Supplementary Table S1). Bland-Altman plots in test-retest baseline MRI biomarker data (Fig. 2C) showed that measurement error was only constant over the range of biomarker values for median *IAUC*_60_. Consequently, all biomarkers underwent a logarithmic transformation.

### Estimates of biomarker repeatability

**Fig. 3:**
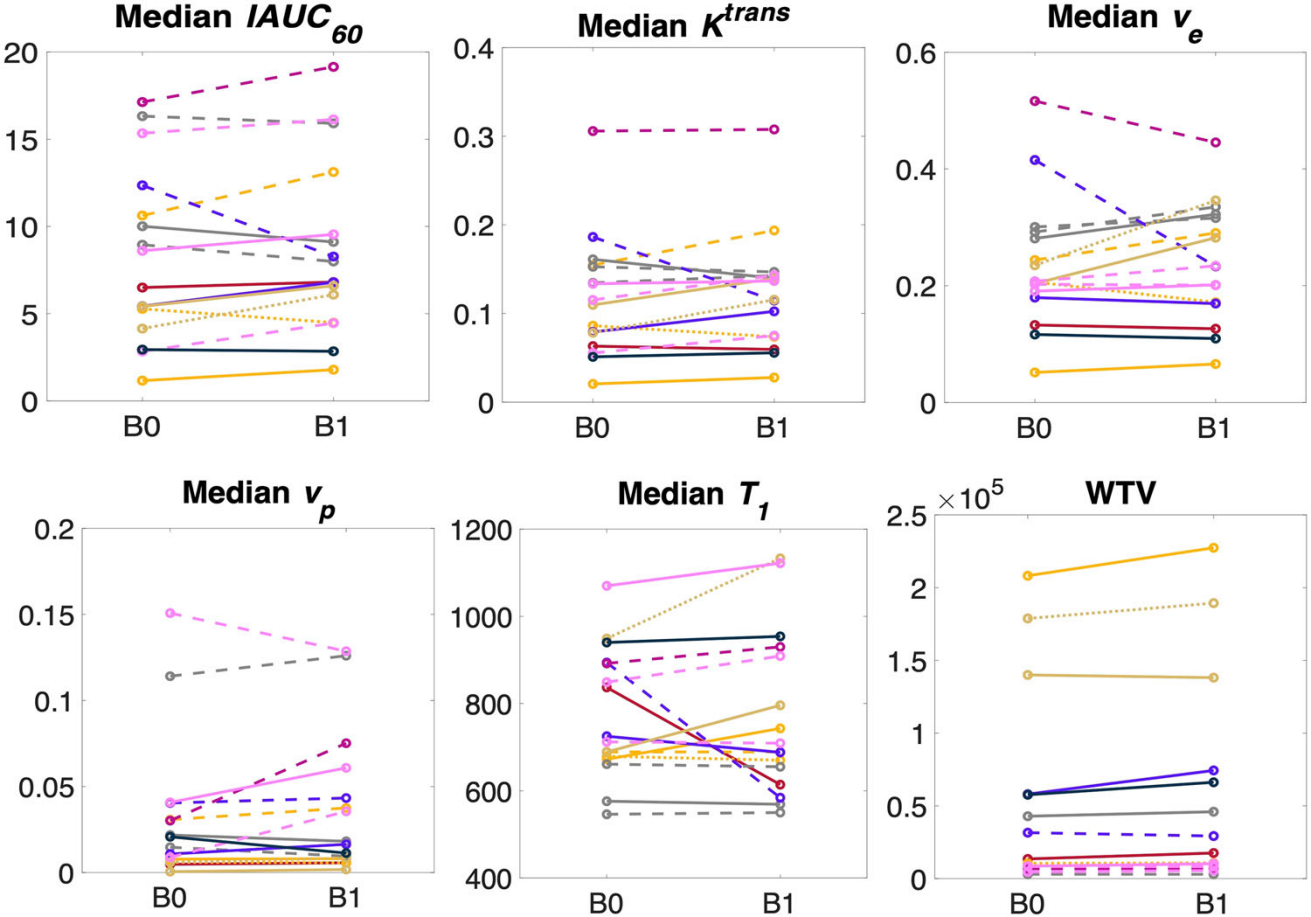
Line plot showing MRI test-retest pre-treatment biomarker values for each lesion. Solid lines represent tumour lesions; dashed lines represent nodal lesions; dotted lines represent metastases

Absolute values of the baseline pre-treatment biomarkers are shown for each parameter in Fig. 3. The wCV ranged from 9.16% for WTV to 42.44% for mean *v*_p_ (Table 2). Asymmetric LOA values are also listed. Data for primary tumours only and nodal lesions only were similar to the repeatability of the overall cohort and are shown in Supplementary Tables S2 and S3).

**Table 2:** Repeatability parameters for each MRI biomarker

Parameter	wCV (%)	95% CI for wCV (%)	Asymmetric LOA (%RC_L_, %RC_U_)
Median *IAUC*_60_	17.02	11.99, 24.24	−37.38, 59.70
Median *K*^trans^	16.45	11.59, 23.42	−36.41, 57.25
Median *v*_*e*_	15.96	11.25, 22.71	−35.55, 55.15
Mean *v*_*p*_	42.44	29.39, 62.66	−67.61, 208.72
Median *T*_1_	10.51	7.42, 14.91	−25.20, 33.70
WTV	9.16	6.47, 12.98	−22.37, 28.82

### Biomarker estimates of cohort mean treatment effect with mixed-effects modelling

The cohort-level change from baseline to week 2 showed significant change with an increase in median *IAUC*_60_, median *K*^trans^, median *v*_*e*_ and reduction in WTV. In distinction, mean *v*_*p*_ and median *T*_1_ showed no significant change (Table 3). Data for primary tumours only and nodal lesions only were similar to the overall cohort change data and are shown in Supplementary Tables S4 and S5.

**Table 3:** Cohort-level mean change in MRI biomarkers from baseline to week 2 of treatment

	Original scale median (IQR)		Logarithmic scale mean (SD)			
Parameter	Baseline	Week 2	Baseline	Week 2	Absolute change $({\beta }_{1})$	*p*-value
Median *IAUC*_60_	8.13 (7.12)	10.69 (6.17)	1.95 (0.68)	2.25 (0.61)	0.15 (0.04)	< 0.01
Median *K*^trans^	0.07 (0.04)	0.09 (0.05)	−2.86 (0.58)	−2.56 (0.52)	0.22 (0.05)	< 0.01
Median v_e_	0.23 (0.15)	0.31 (0.1)	−1.54 (0.51)	−1.23 (0.48)	0.16 (0.03)	< 0.01
Mean v_p_	0.02 (0.03)	0.02 (0.03)	−4.04 (1.27)	−4.03 (1.17)	−0.07 (0.14)	0.59
Median *T*_1_	711.5 (223)	705 (179)	6.63 (0.21)	6.62 (0.23)	−0.02 (0.02)	0.68
WTV	10,338.17 (50,701.01)	7258.71 (11,218.01)	9.98 (1.35)	9.39 (1.35)	−0.19 (0.08)	< 0.01

Estimates at the logarithmic scale obtained from a mixed-effects model; *SD* standard deviation; *p*-value: Wald test for null hypothesis of ${\beta }_{1}$ equal to zero

### Identifying individual lesions exhibiting significant treatment effect: asymmetric RC LOA

**Fig. 4:**
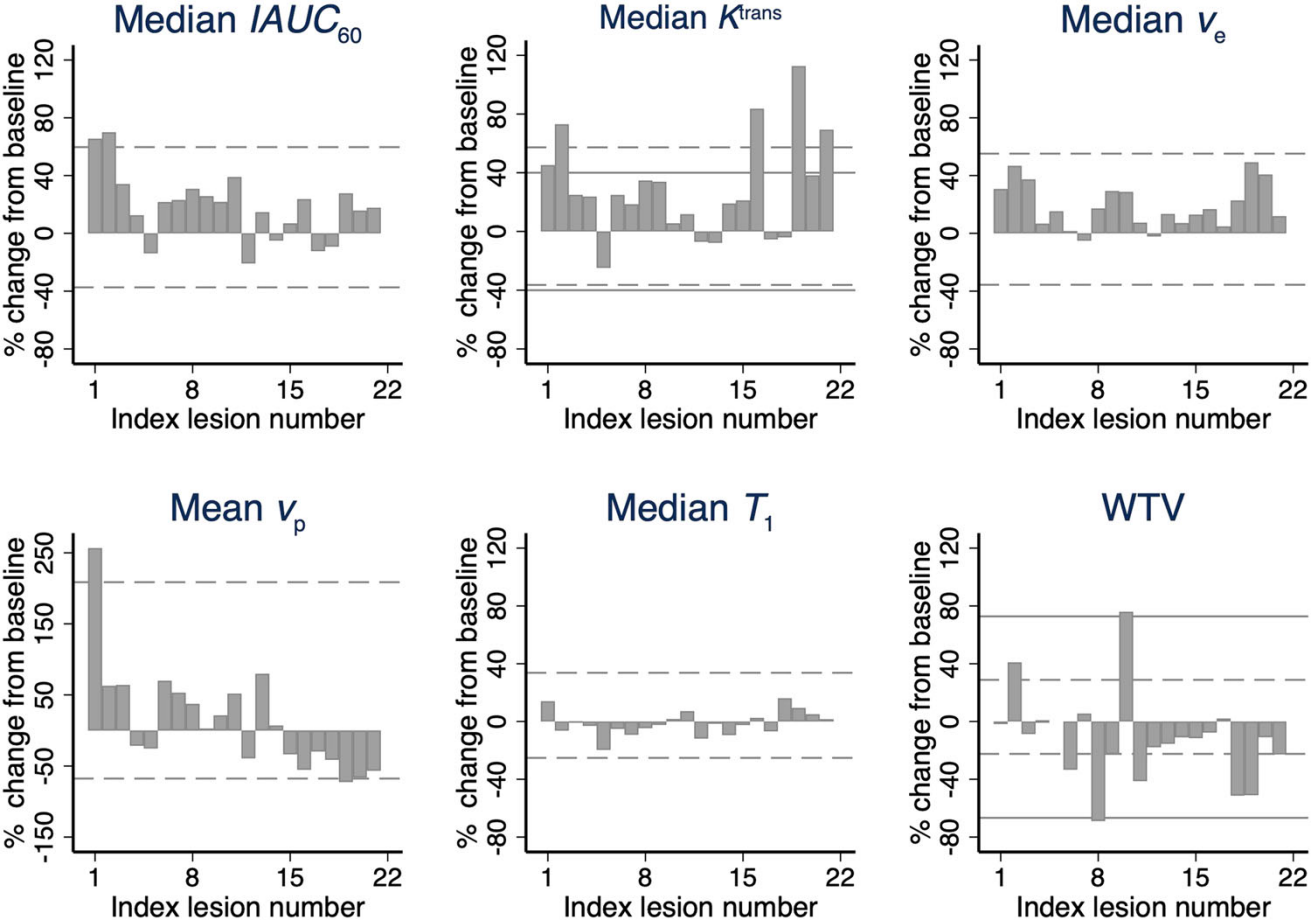
Percentage change in each individual lesion for all MRI parameters. In all cases the asymmetrical (dashed line) LOA are included, parallel to the *x*-axis. Additional literature-based thresholds are added for median *K*^trans^ and WTV (continuous line)

The frequency of each DCE-MRI biomarker showing real change from baseline to week 2, using the asymmetric RC LOA, is shown in Fig. 4. Biomarkers exhibiting cohort-level significant change in mean values had three differing patterns of changes in individual lesions:

- Same-direction change in a minority of lesions: median *IAUC*_60_ and median *K*^trans^ had real increase in two and four individual lesions, respectively, in the same direction as the significant overall cohort effect
- Lack of any change: median *v*_*e*_ did not have real change in any individual lesion, despite a significant overall cohort effect
- Divergent change: WTV showed a significant cohort decrease whereas individual lesions showed diverse real changes with 7/22 reductions and 2/22 increases, while 13/22 lesions did not change beyond the RC LOA.

Finally, biomarkers with no statistical change at the cohort level showed no real change in any individual lesions (median *T*_1_) or just one lesion with change (mean *v*_*p*_).

### Identifying individual lesions exhibiting significant treatment effect: literature thresholds

The frequency of lesions showing real change was compared for literature threshold methods versus asymmetric RC LOA methods. The literature threshold for *K*^trans^ of 40% change to indicate a biological response was close to the asymmetric RC LOA estimate. Therefore, a similar number of lesions (here, 5 rather than 4) were deemed to exhibit real change.

However, for WTV the RECIST-based assessment of size change was substantially greater than the asymmetric RC LOA (−65.7% and +72.8% compared to −22.37% and +28.82%). Consequently, RECIST-based assessment meant that only one lesion had real change—a reduction—differing substantially from the asymmetric RC LOA method of assessment and contrary to the cohort direction of change.

## Discussion

In this study, we sought to define DCE-MRI biomarker precision by measuring repeatability. We also sought to determine if repeatability data could enable statistical analysis of biological response on a per-lesion level, augmenting traditional cohort-based evaluation of treatment effects.

We used a small but representative functional imaging dataset to address these questions. All MRI biomarker data were either non-normally distributed, prone to measurement error dependent on the magnitude of the biomarker, or both. This required data logarithmic transformation and determined which statistical analyses were appropriate for repeatability assessment. Our findings can be summarised in four key messages.

Firstly, MRI repeatability was reported using wCV and %RC values to enable literature comparison. The most commonly cited biomarker *K*^trans^ had a wCV of 16.45% which is comparable to literature wCV values of 20.3% in pelvic tumours of gynaecological origin [28], 11.0% and 20.1% in prostate tumours [29, 30], 15.6% in primary renal cancer [31], and 15.4% and 24% in colorectal liver metastases [18, 32]. This suggests that *K*^trans^ repeatability is acceptable in patients with NSCLC with thoracic lesions. These wCV values are similar to those obtained from biomarkers from other imaging modalities such as [^18^F]FDG PET-CT SUV_max_ in multiple studies [33].

Secondly, repeatability can be compared between different biomarkers derived from the same data with equal constraints (such as size variation and tumour motion). We measured DCE-MRI biomarkers including model-free *IAUC*_60_ and extended Tofts model parameters (*K*^trans^, *v*_*e*_, *v*_*p*_), as well as native *T*_1_ and WTV. We found negligible differences in the precision of *IAUC*_60_ and *K*^trans^—the two most widely used DCE-MRI biomarkers—consistent with some literature [34] despite other studies reporting two-fold differences [32]. As with previous studies [18, 35] we found that WTV was highly repeatable with WTV of less than 10% wCV but that *v*_p_ precision was very poor, approaching 50% wCV.

Thirdly, cohort-level analysis can identify biomarkers that change with therapy and mixed-effects modelling increases data inclusion in studies where patients have variable numbers of lesions. Since cohort changes were significant for some MRI biomarkers (median *IAUC*_60_, median *K*^trans^, median *v*_*e*_ and WTV) but not others (mean *v*_p_ and median *T*_1_), even small studies can identify the best candidate biomarkers for use in subsequent larger studies.

Fourthly, repeatability data provides complimentary information on biological response by identifying the frequency of real change in individual lesions. Our data show that some biomarkers (here, *v*_*e*_) can exhibit significant cohort change in the absence of any individual lesion changing beyond the asymmetric RC LOA. In distinction, other biomarkers (here, median *IAUC*_60_ and median *K*^trans^) exhibit significant cohort change as well as showing significant changes in some individual lesions. Furthermore, other biomarkers (here, WTV) can show significant change at the cohort level but have individual lesions that change in variable manner (increase, no change or decrease). Finally, different methods exist for identifying which lesions exhibit real change (literature thresholds instead of asymmetric RC LOA) and the choice of method can substantially alter the interpretation of the frequency of real change in a cohort.

Limitations of the study include analysing one dataset and using single-centre data, but these limitations are typical to most studies in the literature that deploy DCE-MRI biomarkers. Specific findings will vary from those from other small studies but the principles—that data need to be evaluated for normality, that precision can be measured and can improve evaluation of biological response—are generalisable. In theory data from a research study such as presented here could be extrapolated to patients undergoing functional imaging as part of routine clinical practice. However, further work is required to determine if other authors obtain similar data to that reported here.

Biomarker studies that detect early pathophysiological change following therapy may offer insight into personalised therapy. However, the predictive relationship between this biomarker change and subsequent clinical response must be further evaluated in larger studies.

Collectively, data from our study suggest that measuring biomarker repeatability not only provides useful information regarding precision but also advances the understanding of early pathophysiological changes in the tumour microenvironment.

## Supplementary information

Supplementary Material

## Abbreviations

DCE-MRI: Dynamic contrast-enhanced MRI
FDG: Fluorodeoxyglucose
IAUC60: Initial area under the gadolinium contrast agent concentration-time curve at 60 seconds
*K*^trans^: Volume transfer constant
LOA: Limits of agreement
MRI: Magnetic resonance imaging
NSCLC: Non-small cell lung cancer
PET: Positron emission tomography
RC: Repeatability coefficient
RECIST: Response evaluation criteria in solid tumours
ROI: Region of interest
SUV_max_: Maximum standardised uptake value
*v*_*e*_: Fractional volume of the extravascular extracellular space
*v*_*p*_: Fractional blood plasma volume
wCV: Within-subject coefficient of variation
WHO: World Health Organisation
WTV: Whole tumour volume
