# Supplementary material for: Measuring repeatability of dynamic contrast-enhanced MRI biomarkers improves evaluation of biological response to radiotherapy in lung cancer
Source: Eur Radiol. 2024 Aug 9;35(2):664–73. doi: 10.1007/s00330-024-10970-7 (PMC11782379; doi:10.1007/s00330-024-10970-7)
Supplement: Supplementary file 1 — Supplementary Material [file 330_2024_10970_MOESM1_ESM.pdf]

Measuring repeatability of dynamic contrast-enhanced MRI biomarkers improves evaluation of biological response to radiotherapy in lung cancer

ELECTRONIC SUPPLEMENTARY MATERIAL

Supplementary materials and methods

Assessing biomarker repeatability – model specification

Biomarker repeatability refers to the magnitude of its measurement error under a set of repeatable conditions and was estimated following recommendations by the Quantitative Imaging Biomarker Alliance. In our study, for patients with paired baseline scans, we denote Y_{ijk} as the measurement of any DCE-MRI quantitative biomarker for lesion j $(j = 1,..n_i)$ of subject i $(i = 1,2 \ldots N)$ at scan k $(k = 1,2)$.

Bland-Altman plots of the mean of the replicates for each lesion in the X-axis $((Y_{ij1} + Y_{ij2})/2)$ and the difference between them in the Y-axis $(Y_{ij1} - Y_{ij2})$ can be used to illustrate trends in the variability of the biomarker over the range of measurement values. When the variability is not constant, a logarithmic transformation of the data may be required. This variability is designated as within-lesion variability (noted below by σ_w^2), and repeatability metrics are derived from it.

Two further sources of variability should be accounted for in our setting: (i) the variability between any two lesions within the same patient, referred to as within-patient lesion variability (σ_{wb}^2); and (ii) the variability from one patient to another, or between-subject variability (σ_b^2). The following random effects model is assumed for each measurement of the biomarker:

$$Y_{ijk} = \mu + u_i + v_{ij} + \varepsilon_{ijk}. \quad (1)$$

Where μ is the overall mean across measurements for the biomarker, u_i and v_{ij} are the random contribution to the mean from the ith subject, and their j^{th} lesion, respectively, and are assumed to be distributed as $N(0, \sigma_b^2)$ and $N(0, \sigma_{wb}^2)$, respectively. ε_{ijk} is the measurement error term for each observation which is assumed to follow $N(0, \sigma_w^2)$ with σ_w^2 constant for all replications. Estimation of all parameters in model (1) permit estimation of the following repeatability metrics.

When data is distributed normally and the within-lesion variance can be assumed constant across the value of the biomarker, $RC = 1.96\sqrt{2\hat{\sigma}_w^2} = 2.77\hat{\sigma}_w$, where $\hat{\sigma}_w^2$ is the estimated σ_w^2.

RC will be then expressed in the original units of the biomarker. Alternatives for data not distributed normally are described in the main text.

Estimating cohort mean treatment effect with mixed effects modelling – model specification

For each biomarker, response to therapy was examined in the cohort by calculating percentage change from baseline to week 2 (on the log-transformed data), by adapting the mixed effects model in equation (1). Here, random effects continue to accommodate clustering of lesions within the patients with the timepoints at which the scans were conducted (pre-treatment, week 2) incorporated as fixed effects. The equation of the mixed effects model is now given by,

$$Y_{ijk} = \beta_0 + \beta_1 x_{ijk} + u_{0i} + u_{1ij} + \varepsilon_{ijk} \tag{2}$$

where Y_{ijk} is the measurement of the MRI biomarker for the jth lesion of the ith patient at the kth timepoint, with x_{ijk} being the time indicator (0 for baseline, 1 for week 2). Therefore, β_0 and β_1 represent the mean biomarker value baseline and the mean change at week 2, respectively. u_{0i}, u_{1ij} are the random effects intercepts for the ith patient and their j^{th} lesion, respectively, and ε_{ijk} is the measurement error, all distributed similarly to model (1) above. A significant treatment effect over time is determined when the parameter β_1 is estimated to be significantly different from zero.

Identifying individual lesions exhibiting significant treatment effect

In RECIST 1.1, a 30% change is considered to indicate reduction in a tumour in one dimension. Therefore, a 3D equivalent is where a tumour measures 0.7 x 0.7 x 0.7 of its original volume = 0.343 if the lesion can be approximated to a sphere. This means that a reduction of 1-0.343 = 0.657 (or 65.7%) is required to indicate 'real decrease' in volume.

Further, progression due to size increase is 20%. Therefore, a 3D equivalent is where a tumour measures 1.2 x 1.2 x 1.2 of its original volume = 1.728 if the lesion can be approximated to a sphere. This means that an increase of 1.728-1 = 0.728 (or 72.8%) is required to indicate 'real increase' in volume.

Supplementary Table S1: *Descriptive summary of the cohort parameter values for each of the six MRI biomarkers and normality tests*

	Baseline		Week 2	
**Parameters**	**Shapiro-Wilks test p-value**	**Mean ± SD Median (IQR)**	**Shapiro-Wilks test p-value**	**Mean ± SD Median (IQR)**
Median $IAUC_{60}$	0.07	8.86± 4.77<br>8.13(7.12)	0.49	10.10± 5.47<br>10.69(6.17)
Median K^{trans}	0.004	0.07 ± 0.03<br>0.07(0.04)	0.82	0.09±0.04<br>0.09(0.05)
Median v_e	0.44	0.26±0.11<br>0.23(0.15)	0.82	0.32±0.12<br>0.31(0.10)
Mean v_p	<0.001	0.03±0.04<br>0.02 (0.03)	0.003	0.03±0.03<br>0.02(0.03)
Median T_1	0.03	777.76±164.15<br>710.5(236)	0.17	767.38±177.89<br>705(179)
Whole tumour volume	<0.001	44310.57±63969.47<br>10338.17 (50701.01)	<0.001	33083.18±59365.46<br>7258.71(11218.01)

SD: Standard Deviation; IQR: interquartile range, defined as Q3 (75% percentile)-Q1 (25% percentile)

Supplementary Table S2: *Repeatability parameters for each MRI biomarker within primary lesions (N=7)*

Parameter	wCV (%)	95% CI for wCV (%)	Asymmetric LOA ($\%RC_L$, $\%RC_U$)
**Median $IAUC_{60}$**	14.72	8.69, 25.10	-33.34, 50.01
**Median K^{trans}**	13.35	7.89, 22.73	-30.81, 44.52
**Median v_e**	11.98	7.08, 20.36	-28.16, 39.19
**Mean v_p**	40.69	23.49, 74.00	-66.18, 195.69
**Median T_1**	9.74	5.76, 16.52	-23.61, 30.90
**WTV**	11.79	6.97, 20.03	-27.78, 38.46

Supplementary Table S3: *Repeatability parameters for each MRI biomarker within nodal lesions (N=7)*

Parameter	wCV (%)	95% CI for wCV (%)	Asymmetric LOA ($\%RC_L$, $\%RC_U$)
**Median *IAUC*$_{60}$**	17.86	10.39, 31.00	-38.79, 63.37
**Median K^{trans}**	17.67	10.42, 30.28	-38.49, 62.57
**Median v_e**	15.70	9.85, 25.16	-35.10, 54.08
**Mean v_p**	49.17	28.09, 92.40	-72.44, 262.87
**Median T_1**	11.62	6.87, 19.75	-27.44, 37.83
**WTV**	7.08	4.19, 11.98	-17.79, 21.63

Supplementary Table S4: *Cohort-level mean change in MRI biomarkers from baseline to week 2 of treatment in patients with primary lesion (n=11). Estimates at the logarithmic scale obtained from a mixed-effects model; SD: standard deviation; p-value: Wald test for null hypothesis of* β_1 equal to zero.

	**Original scale Median (IQR)**		**Logarithmic scale Mean (SD)**			
**Parameter**	**Baseline**	**Week 2**	**Baseline**	**Week 2**	**Absolute change (β_1)**	**p-value**
**Median *IAUC*$_{60}$**	6.80(4.33)	10.30(5.31)	1.79(0.66)	2.09(0.60)	0.13 (0.05)	<0.01
**Median K^{trans}**	0.06(0.05)	0.09(0.05)	-2.99(0.67)	-2.62(0.59)	0.20(0.07)	<0.01
**Median v_e**	0.19(0.20)	0.29(0.29)	-1.68(0.61)	-1.38(0.62)	0.14 (0.03)	<0.01
**Mean v_p**	0.02(0.02)	0.01(0.02)	-4.63(1.22)	-4.49(1.11)	-0.07 (0.19)	0.69
**Median T_1**	743(268)	762(176)	6.64(0.22)	6.66(0.22)	0.002(0.02)	0.93
**WTV**	42892.4(65768.34)	15287.29(44652.08)	10.82(1.08)	10.27(1.38)	-0.11 (0.09)	0.21

Supplementary Table S5*: Cohort-level mean change in MRI biomarkers from baseline to week 2 of treatment in patients with nodal lesion (n=8). Estimates at the logarithmic scale obtained from a mixed-effects model; SD: standard deviation; p-value: Wald test for null hypothesis of* β_1 equal to zero.

	**Original scale Median (IQR)**		**Logarithmic scale Mean (SD)**			
**Parameter**	**Baseline**	**Week 2**	**Baseline**	**Week 2**	**Absolute change (β_1)**	**p-value**
**Median $IAUC_{60}$**	13.12(7.86)	13.87(6.11)	2.39(0.54)	2.58(0.53)	0.16 (0.06)	<0.01
**Median K^{trans}**	0.07(0.04)	0.10(0.05)	-2.62(0.45)	-2.37(0.44)	0.23(0.09)	<0.01
**Median v_e**	0.29(0.13)	0.36(0.07)	-1.22(0.30)	-1.01(0.17)	0.19 (0.05)	<0.01
**Mean v_p**	0.04(0.08)	0.06(0.05)	-3.13(0.94)	-3.15(0.85)	-0.10(0.24)	0.66
**Median T_1**	708(237)	694.5(265)	6.58(0.18)	6.56(0.23)	-0.03(0.03)	0.42
**WTV**	6928.77(2749.51)	4784.15(1814.68)	8.91(0.66)	8.51(0.35)	-0.39(0.13)	<0.01
